# Purification, Structural Analysis and Cardio-Protective Activity of Polysaccharides from Radix Astragali

**DOI:** 10.3390/molecules28104167

**Published:** 2023-05-18

**Authors:** Shilei Wang, Yuan Peng, Yixin Zhuang, Nan Wang, Jianchang Jin, Zhajun Zhan

**Affiliations:** 1 College of Biology and Environmental Engineering, Zhejiang Shuren University, Hangzhou 310015, China; 2 College of Pharmaceutical Science, Zhejiang University of Technology, Hangzhou 310014, China

**Keywords:** *Astragalus membranaceus*, polysaccharide, cardio-protection, antioxidant

## Abstract

Two polysaccharides, named APS2-I and APS3-I, were purified from the water extract of Radix Astragali. The average molecular weight of APS2-I was 1.96 × 10^6^ Da and composed of Man, Rha, GlcA, GalA, Glc, Gal, Xyl, and Ara in a molar ratio of 2.3:4.8:1.7:14.0:5.8:11.7:2.8:12.6, while the average molecular weight of APS3-I was 3.91 × 10^6^ Da and composed of Rha, GalA, Glc, Gal, and Ara in a molar ratio of 0.8:2.3:0.8:2.3:4.1. Biological evaluation showed APS2-I and APS3-I had significant antioxidant activity and myocardial protection activity. Furthermore, total polysaccharide treatment could significantly enhance hemodynamic parameters and improve cardiac function in rat ischemia and reperfusion isolated heart models. These results provided important information for the clinical application of APS in the field of cardiovascular disease and implied that Astragalus polysaccharides (APS) could be considered as a reference for the quality control of Radix Astragali.

## 1. Introduction

Cardiovascular diseases (CVDs) have become the number one killer of people. According to the World Health Organization, about 17.9 million people died from cardiovascular disease in 2019, accounting for 32% of global deaths [1]. Many efforts have been made towards drug development for treating cardiovascular diseases. To date, calcium channel blockers and angiotensin-converting enzyme inhibitors have been widely used in the clinic to treat cardiovascular diseases [2]. However, these medicines have many shortcomings, such as side effects, long treatment cycles, and so on. It is of great significance to find the use of alternative medicines for treating cardiovascular diseases.

*Astragalus membranaceus*, widely distributed all over the world, is a perennial herbaceous plant of the Leguminosae family [3,4,5]. The dried root of *A. membranaceus*, known as Huangqi in China and Radix Astragali in Latin, is a traditional Chinese herbal medicine used to treat many diseases, especially cardiovascular diseases [6,7]. Pharmacological studies showed that the root extract of *A. membranaceus* could improve cardiac function and reduce myocardial cell death [8]. In China, *Astragalus* injection has been widely used to treat cardiovascular disorders with promising clinical effects and no obvious side effects [9]. Phytochemical studies on this plant showed that its bioactive components mainly included polysaccharides, astragalosides, flavonoids, alkaloids, and a variety of trace elements, among which astragalosides and polysaccharides were considered to be the most important components [7,10]. Astragaloside IV is one of the main pharmacological active ingredients of astragalosides, which have a strong protective effect against ischemia reperfusion injury [11]. Herein, the Chinese Pharmacopoeia (2010 version) lists astragaloside IV as a quality-control marker component of Radix Astragali.

Astragalus polysaccharide (APS) is a kind of water-soluble heteropolysaccharide with a weight-average molecular weight (Mw) range of 8.7–4800 kDa. So far, 24 polysaccharides have been reported from the roots of *A. membranaceus* [12]. The monosaccharide compositions of these polysaccharides mainly include mannose (Man), rhamnose (Rha), glucose (Glc), galactose (Gal), xylose (Xyl), arabinose (Ara), fucose (Fuc), fructose (Fru), and ribose (Rib), and only a small fraction of APS contains glucuronic acid (GlcA) and galacturonic acid (GalA) [12]. Pharmacological studies revealed that APS had anti-atherosclerosis [13,14], anti-inflammatory [15,16,17], anti-diabetes [18,19,20,21], antioxidative [22,23,24], immunomodulatory [25,26,27,28], antitumor [29,30,31,32], and antiviral [33,34,35] effects. Although the pharmacological effects of APS have been extensively studied, most of them focus on immune modulating, antitumor and anti-diabetes. However, information on the cardio-protective effect of APS is still limited. It is generally believed that astragalosides are the main bioactive constituents in Radix Astragali responsible for its clinical use in treating cardiovascular diseases. But it remains unclear whether APS is also a bioactive constituent of Radix Astragali with potential applications in the cardiovascular field.

The primary aim of this study was to investigate whether APS also exhibits cardio-protective properties and identify which homogeneous polysaccharides are responsible for this effect. To achieve this, we evaluated the effects of APS in rat hearts subjected to hypoxia/ischemia and attempted to isolate total polysaccharides to obtain homogenous fractions for further experimentation. Additionally, we conducted molecular weight measurements and in vitro antioxidant activity assays on the purified homogeneous polysaccharides. Furthermore, we utilized a CoCl_2_-induced hypoxia model in H9c2 cells to evaluate the cardio-protective activity of the polysaccharides.

## 2. Results and Discussion

### 2.1. Extraction and Purification of APS2-I and APS3-I

**Figure 1:**
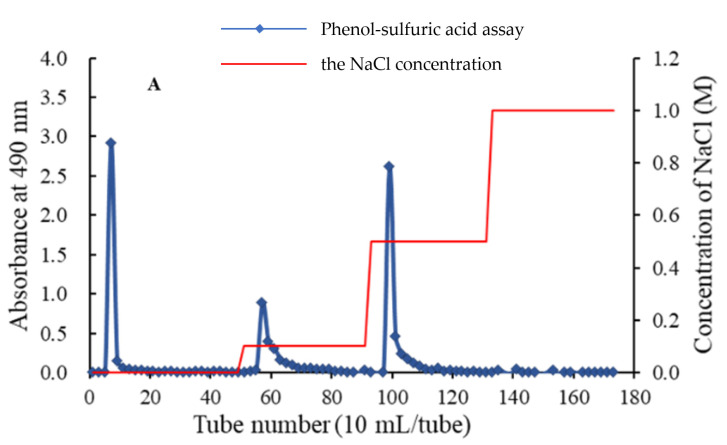

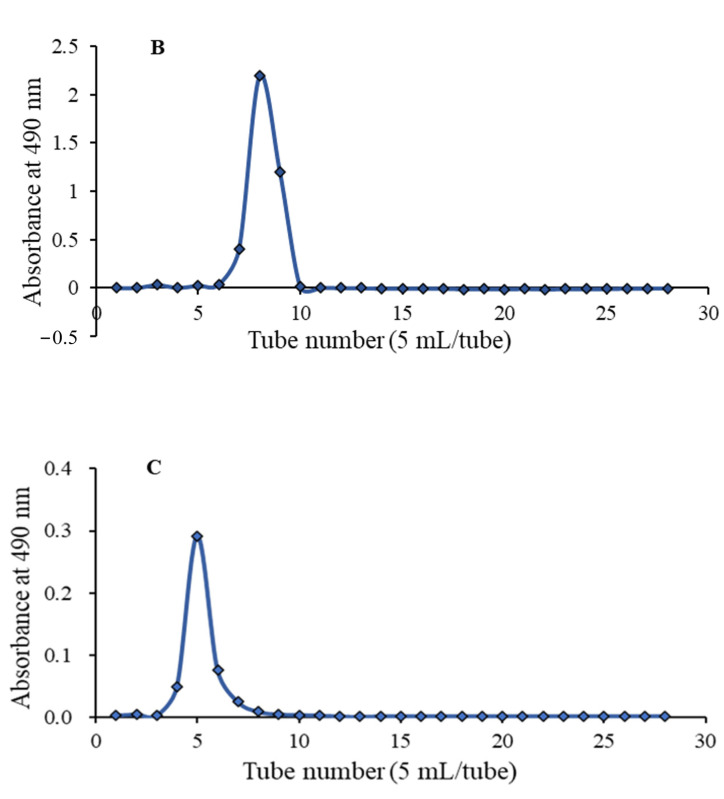
Stepwise elution curve of APS on a DEAE-52 column (**A**) and elution profile of APS2-I (**B**) and APS3-I (**C**) on a Sephadex G-75 column.

The crude APS was obtained from water extract of Radix Astragali through steps of ethanol precipitation and deproteination. Firstly, APS was purified by a DEAE-52 chromatography (Figure 1A), affording three independent elution peaks. Then two fractions, eluted by 0.1 and 0.5 M NaCl solution, were further purified by gel chromatography on a Sephadex G-75 column. As shown in each of Figure 1B,C, a single symmetrical peak was observed, corresponding to APS2-I and APS3-I, respectively.

### 2.2. Determination of Sugar and Protein Contents

The carbohydrate content of the APS was determined as 57.34%, measured by the phenol-sulfuric acid method [36]. UV absorption spectra of APS2-I and APS3-I did not show any absorption peaks in the wavelength 260 to 280 nm, indicating the absence of nucleotides and protein in APS2-I and APS3-I.

### 2.3. Chemical Analysis of APS2-I and APS3-I

From the high-performance gel permeation chromatography (HPGPC) profile, the peaks of APS2-I and APS3-I appeared as a single and symmetrical sharp peak, respectively, indicating that they were all homogeneous polysaccharides. Based on the calibration with standard dextrans, the average molecular weights of APS2-I and APS3-I were estimated to be 1.96 × 10^6^ and 3.91 × 10^6^ Da, respectively.

**Figure 2:**
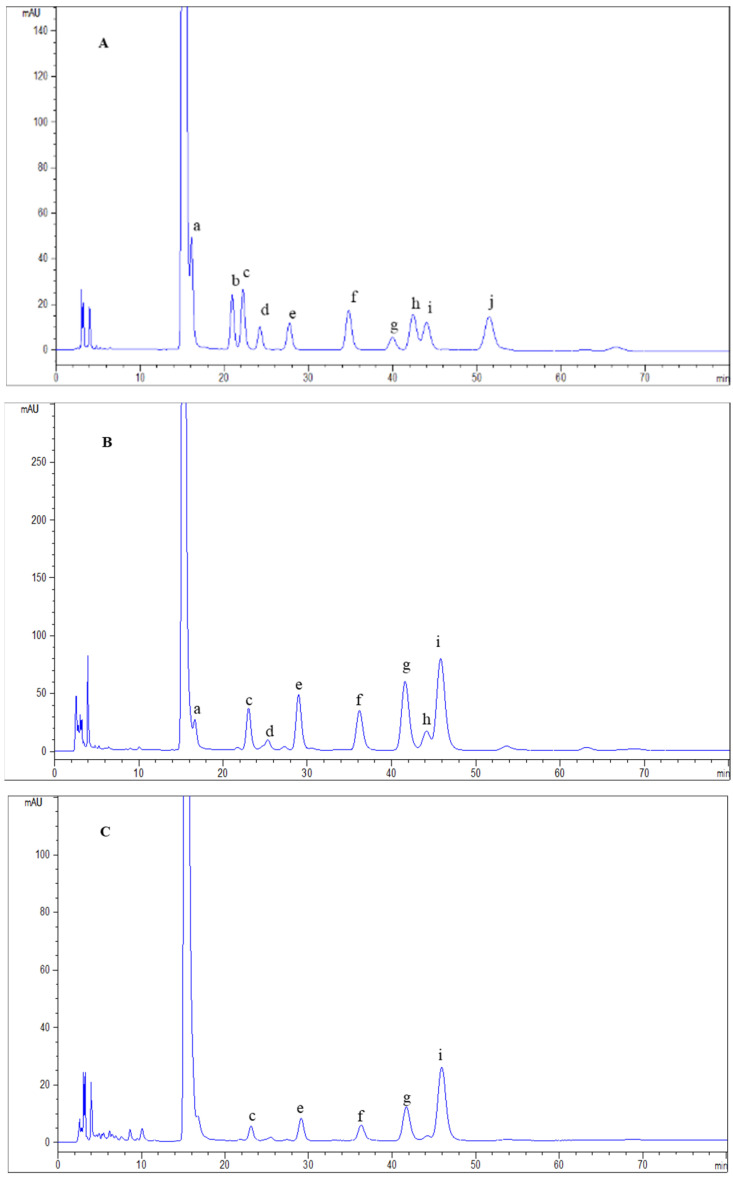
Chromatograms of PMP derivatives of mixed monosaccharide standards (**A**), APS2-I (**B**), and APS3-II (**C**). The mixed standard monosaccharides peaks: a. D-mannose; b. D-ribose; c. L-rhamnose; d. D-glucuronic acid; e. D-galacturonic acid; f. D-glucose; g. D-galactose; h. D-xylose; i. D-arabinose; j. L-fucose.

The monosaccharide compositions of APS2-I and APS3-I were determined by HPLC analysis as shown in Table 1. The HPLC chromatograms of standard monosaccharides are presented in Figure 2. APS2-I was composed of Man, Rha, GlcA, GalA, Glc, Gal, Xyl, and Ara in a molar ratio of 2.3:4.8:1.7:14.0:5.8:11.7:2.8:12.6, which showed that galacturonic acid, galactose and arabinose were the main monosaccharide compositions of APS2-I. APS3-I was composed of Rha, GalA, Glc, Gal, and Ara in a molar ratio of 0.8:2.3:0.8:2.3:4.1, and the main monosaccharide compositions of APS3-I were galactose and arabinose.

The findings showed that APS2-I’s monosaccharide compositions were more complicated than the polysaccharides previously reported from Radix Astragali [12]. APS2-I was composed of eight different monosaccharides including rare GlcA and GalA. GalA was also present in APS3-I. The structures of these two polysaccharides were complex and their molecular weights were both above 1 × 10^6^ Da. The relative molecular mass distribution of polysaccharides from Radix Astragali was very wide (5.6 × 10^3^ Da–7.6 × 10^6^ Da) [37], but most of their molecular weights were below 1 × 10^6^ Da. Therefore, these two polysaccharides may be isolated from Astragalus for the first time The discovery of APS2-I and APS3-I enriches the types of astragalus polysaccharides. However, due to the high molecular weight and poor water solubility of these two polysaccharides, it is difficult to obtain high-quality NMR spectra for further structural analysis.

### 2.4. Antioxidant Activities of APS2-I and APS3-I

**Figure 3:**
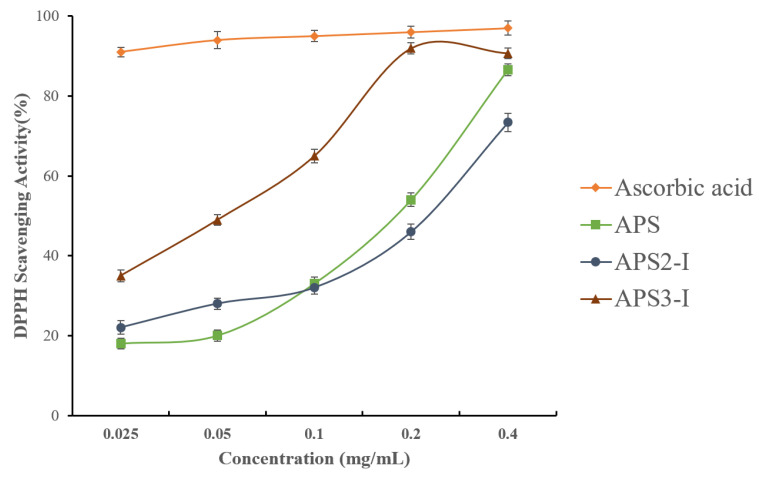
DPPH radical-scavenging activity of APS, APS2-I and APS3-I.

Oxidative stress is one of the major causes of cardiovascular disease. A large body of evidence indicates that reactive oxygen species (ROS) play a detrimental role in cardiovascular disease [38]. The model of scavenging 2,2-diphenyl-1-picrylhydrazyl(DPPH) free radical has been widely used to evaluate the antioxidant activities of natural compounds and has the advantages of convenience and reproducibility [39]. Although the scavenging ability of samples (APS, APS2-I, and APS3-I) was inferior to that of ascorbic acid, they still had strong antioxidant activity. As shown in Figure 3, the DPPH free radical scavenging activity of APS, APS2-I, and APS3-I presented a dose-dependent manner in the range of 0.025–0.4 (mg/mL). At the concentration of 0.4 mg/mL, the scavenging rates of APS, APS2-I, and APS3-I reached 86.61%, 73.43%, and 90.73%, respectively. The IC_50_ values of samples (APS, APS2-I, and APS3-I) were 0.12 mg/mL, 0.15 mg/mL, and 0.04 mg/mL, respectively. APS3-I exhibits higher antioxidant activity than APS2-I, while the crude polysaccharide shows activity levels between those of APS3-I and APS2-I. This indicates that diverse polysaccharides exhibit distinct activities, and the overall activity of crude polysaccharides is a comprehensive result of numerous homogenous polysaccharides. The finding that APS2-I and APS3-I have antioxidant activity raises the possibility that APS can be employed as a natural antioxidant to help treat cardiovascular disease.

### 2.5. Effect of APS on the Cardio-Protection

#### 2.5.1. Protective Effect of APS on CoCl_2_-Induced Hypoxia in H9c2 Cells

**Figure 4:**
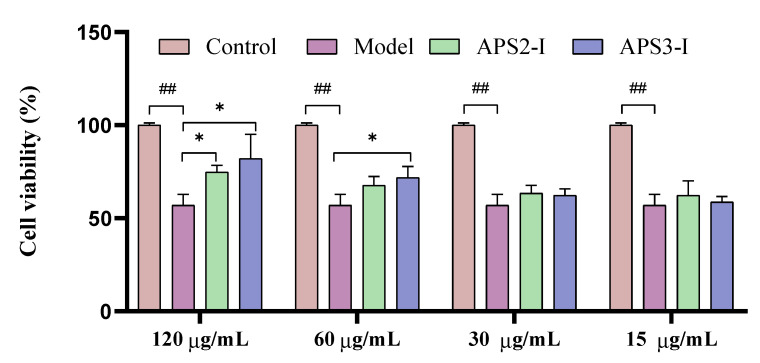
Effects of different concentrations of APS2-I and APS3-I on H9c2 cell viability. (Values are presented as mean ± SD. *n* = 3). ^##^ *p* < 0.01 vs. the control group; * *p* < 0.05 vs. the model group.

Cardiomyocyte hypoxia/ischemia is the primary factor contributing to cardiovascular diseases, and developing an appropriate cell model is essential for conducting in-depth studies of these disorders [40]. It has been reported that cobalt chloride (CoCl_2_) is a common hypoxia mimetic agent widely used in the cell hypoxia model [41]. In this section, CoCl_2_-induced hypoxia in H9c2 model cells was used to determine the effect of APS on the cardio-protection. As shown in Figure 4, CoCl_2_ significantly decreased the cell survival rate of H9c2 cells to 57%, as compared to the control group. After treatment of APS2-I and APS3-I, the cell viability was significantly improved compared with the model group at the concentration of 120 μg/mL and 60 μg/mL, respectively. Furthermore, we found that APS3-I exhibited a better cardio-protection effect than APS2-I. The different effects of APS2-I and APS3-I on cell viability might be due to their differences in monosaccharide composition and molecular weight.

#### 2.5.2. Protective Effect of APS on Isolated Rat Hearts

**Figure 5:**
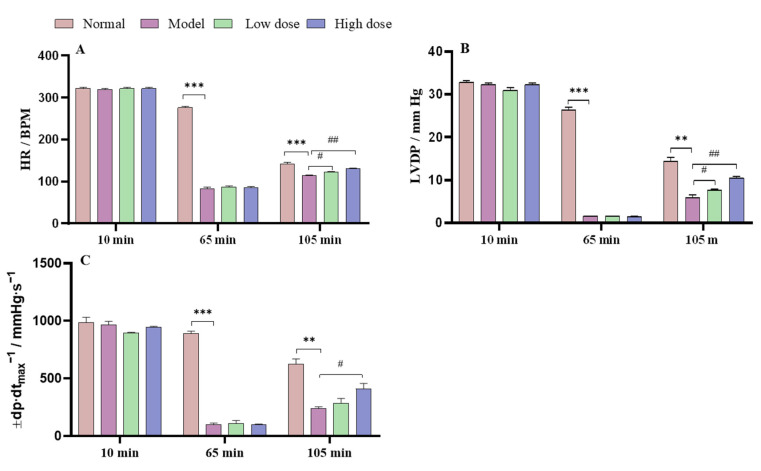
Effects of APS on cardiac function in rats’ hearts subjected to hypoxia/ischemia, (**A**) Effects of APS on heart rate (HR), (**B**) Effects of APS on left ventricular developed pressure (LVDP), (**C**) Effects of APS on maximal left ventricular pressure rising rate (±dp/dt_max_). *** *p* < 0.001 and ** *p* < 0.01 vs. the normal group; ^##^ *p* < 0.01, and ^#^ *p* < 0.05 vs. the model group.

The effect of APS on the parameters for cardiac function in the normal, model, and APS-treated group hearts were recorded and are shown in Figure 5. In the hearts in the model group, hemodynamic parameter (HR, LVDP, and ±dp/dt_max_) recovery rates were significantly decreased compared with those in the normal group after 105 min (reperfusion period). When the isolated rat hearts were irrigated for 10 min, the hemodynamic parameters between the normal, model, and APS groups showed no significant difference. At 65 min of reperfusion, the hemodynamic parameters of the model group were significantly decreased compared with those of the normal group. At 105 min of reperfusion, the APS high-dose group (27.62 mg·L^−1^) significantly improved their hemodynamic parameters compared with the model group and presented a dose-dependent manner.

**Figure 6:**
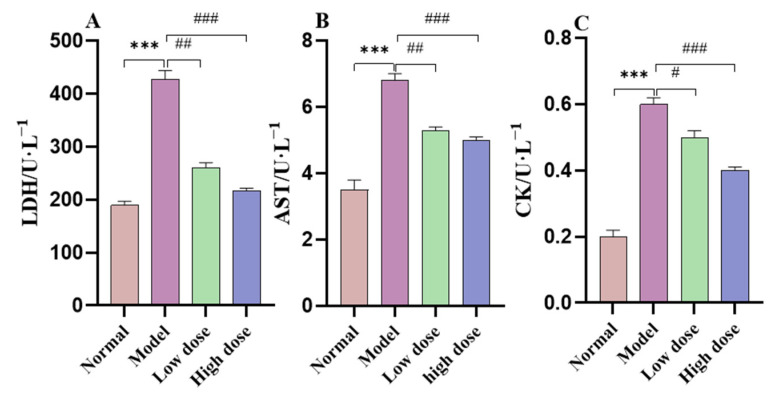
Effects of APS on the levels of (**A**) lactate dehydrogenase (LDH), (**B**) aspartate aminotransferase (AST), and (**C**) creatine kinase isoenzyme (CK) in the effluent of isolated rat hearts after 45 min of reperfusion. (Values are presented as mean ± SD. *n* = 3). *** *p* < 0.001 vs. the normal group; ^###^ *p* < 0.001, ^##^ *p* < 0.01, and ^#^ *p* < 0.05 vs. the model group.

In the physiological state, myocardial enzymes (lactate dehydrogenase (LDH), creatine kinase isoenzyme (CK), and aspartate aminotransferase (AST)) are present in cardiomyocytes, and are released into the blood when the myocardial membrane is damaged by hypoxia/ischemia [42]. Therefore, LDH, CK, and AST levels can sensitively reflect the degree of myocardial injury. At 45 min of reperfusion, the heart effluents were collected. The levels of myocardial tri-enzyme (LDH, AST, and CK) in the model group significantly increased compared with the normal group, as shown in Figure 6. The levels of myocardial tri-enzyme in the APS group significantly decreased compared with the model group.

Research studies have found that reperfusion of blood causes an inflammation-like injury called myocardial ischemia and reperfusion injury (MIRI) [43].

Abnormal levels of cardiac enzymes are indicative of myocardial cell damage and metabolic dysfunction, which is commonly observed in cases of myocardial ischemic injury. CK is almost exclusively present in the heart muscle and has a high sensitivity and specificity, while LDH is abundant in cardiac cells. Upon myocardial injury, the membrane permeability of cells increases, leading to the rapid release and elevation of LDH, AST, and CK, which are specific cardiac enzyme markers [44].

As evident from Figure 6, treatment with APS significantly reduced the levels of tri-enzymes LDH, AST, and CK in a concentration-dependent manner. This reduction was statistically significant compared with the model group, indicating that APS treatment can effectively alleviate MIRI-induced myocardial damage. Therefore, measuring changes in cardiac enzyme levels could serve as an important diagnostic tool for evaluating myocardial ischemic injury severity, and our findings support the potential therapeutic use of APS in treating this condition.

## 3. Materials and Methods

### 3.1. Materials and Chemicals

Water extract of Radix Astragali was provided by Zhejiang Xinguang Pharmaceutical Co., Ltd. (Shengzhou, China). DEAE cellulose-52 and Sephadex G-75 were purchased from Shanghai Yuanye Bio-Technology Co., Ltd. (Shanghai, China). Dextrans with different molecular weights were purchased from Waters Technologies (Milford, MA, USA). 1-Phenyl-3-methyl-5-pyrazolone (PMP), trifluoroacetic acid (TFA), and 2,2-diphenyl-1-picrylhydrazyl (DPPH) were obtained from Shanghai Macklin Biochemistry Corporation (Shanghai, China). Standard monosaccharides (mannose, ribose, rhamnose, glucuronic acid, galacturonic acid, glucose, galactose, xylose, arabinose, and fucose) and ascorbic acid were purchased from Aladdin Reagents Co., Ltd. (Shanghai, China). The H9c2 cardiomyocytes were purchased from Cell Bank of Chinese Academy of Sciences and fetal bovine serum (FBS) was purchased from Hangzhou Sijiqing Co., Ltd. (Hangzhou, China). Cell Counting Kit-8 (CCK-8) and DMEM medium were purchased from Beijing Solarbio Sciences & Technology Co., Ltd. (Beijing, China). The Langendorff’s apparatus (LGF-2) was purchased from Shanghai Wolong Technology Co., Ltd. (Shanghai, China). Assay kits for LDH, CK, and AST were obtained from Nanjing Jiancheng Bioengineering Institute. All other reagents used in this study were of analytical grade.

### 3.2. Preparation of the Total Polysaccharides

The water extract of Radix Astragali was concentrated using a rotary evaporator (RE-3000A, Shanghai Yarong Biochemical Instrument Factory, Shanghai, China) under reduced pressure until no more water was evaporated. Distilled water was then added dropwise until the sample was fully dissolved, and the volume of distilled water used was recorded. Anhydrous ethanol was slowly added dropwise to the saturated aqueous solution of the sample and shaken until the ethanol concentration reached 80% [45]. The mixture was left to stand overnight at 4 °C. After centrifugation at 3000 rpm for 15 min, the sediment was redissolved in an appropriate volume of distilled water and then deproteinized with Sevag reagent (CHCl_3_: *n*-butanol = 4:1, *v*/*v*) three times. Subsequently, the refined polysaccharide was dialyzed (cutoff Mw 5000 Da) against distilled water at room temperature for 48 h. Finally, the retentate portion was concentrated and lyophilized to obtain total polysaccharides (APS).

### 3.3. Determination of Carbohydrate and Protein Contents

The carbohydrate content of APS was determined by the phenol-sulfuric acid method with D-glucose as a standard [46]. The protein contentS of APS2-I and APS3-I were determined using UV-vis absorption spectra (UV-2450, Shimadzu, Kyoto, Japan). The record valve ranged from 200 to 400 nm.

### 3.4. Isolation and Purification of the Total Polysaccharides

The method used for the separation of crude polysaccharides is similar to the one described by Xiuying Pu [47], with slight modifications. The APS (500 mg) was dissolved in 10 mL distilled water, submitted to pre-equilibrated DEAE-52 cellulose column chromatography (3 cm × 80 cm), and eluted with distilled water and 0.1, 0.5, and 1.0 M NaCl solutions, each at a flow rate of 1.0 mL/min. The elution (10 mL/tube) was collected and detected by tracking the absorbance at 490 nm. Two fractions, eluted by 0.1 and 0.5 M NaCl solution, were further purified by a Sephadex G-75 (2 cm × 80 cm) column, which was eluted with 0.1 M NaCl solution at a flow rate of 0.5 mL/min, and elution (5 mL/tube) was detected as described above. The elution was collected, dialyzed, and lyophilized to afford two polysaccharides, named APS2-I and APS3-I.

### 3.5. Homogeneity and Molecular Weight Analysis

The molecular weight analysis method used is also similar to that described by Xiuying Pu [47]. Homogeneity and the molecular weight of polysaccharides were measured using HPGPC with a Shimadzu LC-10A HPLC apparatus equipped with a refractive index detector (RID) and serially linked with three types of columns (a Shodex Sugar KS-805, a Shodex Sugar KS-804, plus a Shodex Sugar KS-803; 7.8 mm × 300 mm). Dextrans (5.2, 11.6, 23.8, 48.6, 148, 273, 410, and 668 kDa) were used to create the standard curve. The sample solution (10 μL, 2 mg/mL) was injected in each run, with water as the mobile phase at 40 °C at a flow rate of 0.8 mL/min.

### 3.6. Monosaccharide Composition Analysis

The monosaccharide compositions were measured by previously reported methods [48], and the PMP-labeled polysaccharide was analyzed by HPLC (Agilent Technologies, Santa Clara, CA, USA). Briefly, the dried samples (5 mg) were hydrolyzed with 2 M trifluoroacetic acid (TFA) at 120 °C for 6 h. After hydrolysis, methanol was repeatedly added to remove trifluoroacetic acid, and the solution was concentrated under reduced pressure and then neutralized with 1 mL NaOH (0.3 mol/L). 400 μL sample hydrolysates and 400 μL PMP-methanol (0.5 mol/L) were added to the test tube, then the mixture was incubated at 70 °C for 100 min. When cooled to room temperature, 500 μL HCl (0.3 mol/L), 300 μL deionized water, and 2 mL chloroform were added, then centrifuged at 3000 rpm for 10 min. The upper water was filtered through a 0.45 μm membrane for HPLC analysis. Standard monosaccharide samples (glucose, galactose, arabinose, mannose, rhamnose, and xylose) were derivatized under the same conditions.

### 3.7. Evaluation of Antioxidant Ability In Vitro

The DPPH radical scavenging activity was measured as reported by Blois [49] with some modifications. Briefly, 1 mL polysaccharide samples (APS, APS2-I, APS3-I) of different concentrations were added to 2 mL of DPPH ethanol solution (0.025 mg/mL). The mixture was shaken vigorously and incubated in the dark for 30 min, and then the absorbance was measured at 517 nm against a blank. Ascorbic acid and anhydrous ethanol were used as positive control and blank control, respectively. The DPPH radical scavenging rate was calculated according to the following equation:

(1) $$\mathrm{DPPH}\;\mathrm{scavenging}\;\mathrm{activity}\;(\%)=1-\left[\frac{A_{i}-A_{j}}{A_{0}}\right]\times 100$$

where A_0_, A_i_, and A_j_ are the absorbance of DPPH solution, mixture, and sample solution, respectively.

### 3.8. Evaluation of Cardio-Protective Activity on H9c2 Cells

The H9c2 cardiomyocytes were grown in DMEM medium containing 10% (*v*/*v*) FBS in an incubator under an atmosphere of 5% CO_2_ at 37 °C. Experiments were performed when cell growth was approximately 80% confluent. Three independent experiments were performed.

H9c2 cells in exponential phase were seeded in 96-well plates at a density of 8000 cells/well. Cells were treated with different concentrations (120 μg/mL, 60 μg/mL, 30 μg/mL, and 15 μg/mL) of polysaccharide samples (APS, APS2-I, and APS3-I) for 24 h. The blank control was added with the same volume of sterile water. Then, the medium was replaced with normal medium containing 400 μM cobalt chloride to simulate hypoxia for 18 h. After all treatments, all wells were added CCK-8 in an incubator at 37 °C for 2 h. The absorbance was measured using a microplate reader (Bio-Rad iMark, Hercules, CA, USA) at 570 nm. Cell viability was calculated by dividing the absorbance of wells containing samples (corrected for background) by the absorbance of wells containing medium alone (corrected for background).

### 3.9. Assay of Cardio-Protective Activity on Rat Isolated Hearts

#### 3.9.1. Animals

All animal experiments were approved by the Animal Ethics Committee of Zhejiang University of Technology. The certificate number of these rats was SCXK2019-0002. Adult male Sprague-Dawley (SD) rats (200 ± 20 g) were obtained from Zhejiang Academy of Medical Sciences (Hangzhou, China), and housed at a constant temperature of 25 ± 2 °C under a 12 h light-dark photoperiod.

#### 3.9.2. Rat Langendorff-Perfused Isolated Heart Preparation

Langendorff-perfused isolated heart recordings were carried out by a previously reported method [50]. Briefly, rats were anesthetized with sodium pentobarbital (60 mg/kg). Each heart was rapidly excised by thoracotomy, and the aorta was cannulated. The isolated heart was quickly removed and arrested in Krebs-Henseleit (K-H) perfusion buffer (4 °C). Then, the heart was mounted on the Langendorff apparatus (LGF-2) by inserting a perfusion cannula into the aorta. A fluid-filled latex balloon was inserted into the left ventricle via the left auricle. The latex balloon was connected to a pressure transducer to continuously monitor the changes in left ventricle pressure. Isolated rat hearts were randomly assigned into four groups (*n* = 3): normal group, model group, and two APS-treated groups (APS low-dose group, pretreated with 20.72 mg/L total polysaccharides; and APS high-dose group, pretreated with 27.62 mg/L of total polysaccharides). The normal group was continuously perfused for 110 min. The hearts in the model group and the APS treatment groups were perfused with buffer to stabilize them for 30 min, followed by no-flow global ischemia for 30 min. Then, the isolated hearts were subjected to 50 min of reperfusion with either K-H buffer or K-H buffer containing APS. APS was diluted with K-H solution to the required final concentration.

#### 3.9.3. Quantification of Myocardial Injury

The hemodynamic parameters, including heart rate (HR), left ventricular developed pressure (LVDP) and ventricular contractility (±dP/dt) were measured by a computer-based data acquisition system (PTB4868, ADInstruments, Sydney, Australia).

The protein expression of LDH, CK, and AST in the perfusate was measured as the degree of cardiac injury. LDH, CK, and AST were collected from the coronary effluent after the reperfusion period, and the levels of LDH, CK, and AST in the effluent were detected by using commercially available detection kits.

### 3.10. Statistical Analysis

Data are expressed as the mean ± standard deviation (SD) for three replicates. All statistical analyses were performed using GraphPad Prism software (version 8.0.1). A value of *p* less than 0.05 was considered statistically significant.

## 4. Conclusions

To investigate whether APS has cardio-protective properties, its effects on cardiac function were tested in rat hearts subjected to hypoxia/ischemia. The results of our study indicate that treatment with total polysaccharides can significantly improve both the contractile and diastolic functions of the myocardium, while reducing the release of hypoxia/ischemia-induced tri-enzymes LDH, AST, and CK into the blood in a rat Langendorff-perfused isolated heart model. These findings suggest that APS is also a bioactive compound in Radix Astragali with potential cardio-protective effects. Identifying the homogeneous polysaccharides that exhibit cardio-protective activity would hold significant scientific importance. Therefore, we tried to isolate the total polysaccharides to obtain homogeneous polysaccharides for further experimentation. Through isolation and purification, we obtained two homogeneous polysaccharides, named APS2-I and APS3-I.

Based on the calibration with standard dextrans, APS2-I and APS3-I were found to have average molecular weights of 1.96 × 10^6^ Da and 3.91 × 10^6^ Da, respectively. APS2-I had a molar ratio of Man, Rha, GlcA, GalA, Glc, Gal, Xyl, and Ara of 2.3:4.8:1.7:14.0:5.8:11.7:2.8:12.6. The main monosaccharides in APS2-I were galacturonic acid, galactose, and arabinose. In contrast, APS3-I had a molar ratio of Rha, GalA, Glc, Gal, and Ara of 0.8:2.3:0.8:2.3:4.1, with galactose and arabinose being the primary monosaccharide components. The relative molecular mass distribution of polysaccharides from Radix Astragali is very wide (5.6 × 10^3^ Da–7.6 × 10^6^ Da), but most of their molecular weights were below 1 × 10^6^ Da. Therefore, these two polysaccharides may be isolated from Astragalus for the first time.

However, the yields of APS2-I and APS3-I were low, and we were only able to accumulate a few milligrams through repeated isolation. As a result, we were unable to perform animal studies to evaluate the cardio-protective properties of these homogeneous polysaccharides. The DPPH free-radical-scavenging activity of APS, APS2-I, and APS3-I was investigated in a dose-dependent manner within the range of 0.025–0.4 (mg/mL). The IC_50_ values for APS, APS2-I, and APS3-I were 0.12 mg/mL, 0.15 mg/mL, and 0.04 mg/mL, respectively. The results suggest that APS3-I has higher antioxidant activity than APS2-I, while the crude polysaccharide showed activity levels between those of APS3-I and APS2-I. The discovery of antioxidant activity in APS2-I and APS3-I highlights the potential of APS as a natural antioxidant for treating cardiovascular disease. CoCl_2_-induced hypoxia in H9c2 cells was used to evaluate the cardio-protective effects of APS. Treatment with APS2-I and APS3-I resulted in significant improvements in cell viability compared with the model group, with APS3-I being more effective at concentrations of 120 μg/mL and 60 μg/mL.

In conclusion, APS shows promise as a new source of natural antioxidant and cardio-protective compounds. Further investigation is warranted to elucidate the mechanisms underlying these effects and explore the potential clinical applications of APS in cardio-protection. In addition, the discovery of APS as an active component suggests that it could serve as a reference for quality control in Radix Astragali.

## Figures and Tables

**Table 1:** The monosaccharide composition of APS2-I and APS3-I.

Sample	Monosaccharide Composition in a Molar Ratio							
	Man	Rha	GlcA	GalA	Glc	Gal	Xyl	Ara
APS2-I	2.3	4.8	1.7	14.0	5.8	11.7	2.8	12.6
APS3-I	nd ^1^	0.8	nd	2.3	0.8	2.3	nd	4.1

^1^ nd: not detected.

## Data Availability

Data sharing not applicable.
